# ^18^F-fluorothymidine (FLT)-PET and diffusion-weighted MRI for early response evaluation in patients with small cell lung cancer: a pilot study

**DOI:** 10.1186/s41824-019-0071-5

**Published:** 2020-01-27

**Authors:** Tine Nøhr Christensen, Seppo W. Langer, Katrine Engholm Villumsen, Helle Hjorth Johannesen, Johan Löfgren, Sune Høgild Keller, Adam Espe Hansen, Andreas Kjaer, Barbara Malene Fischer

**Affiliations:** 1 Department of Clinical Physiology, Nuclear Medicine & PET, Rigshospitalet, University of Copenhagen, Blegdamsvej 9, 2100 Copenhagen Ø, Denmark; 2 Cluster for Molecular Imaging, University of Copenhagen, Copenhagen, Denmark; 3 Department of Oncology, Rigshospitalet, University of Copenhagen, Copenhagen, Denmark; 4 PET Centre, School of Biomedical Engineering and Imaging Science, Kings College London, London, UK

**Keywords:** Small cell lung cancer, SCLC, FLT-PET, ^18^F-fluorothymidine, PET/MRI, Diffusion-weighted MRI, DW-MRI, Early treatment evaluation, Response evaluation, Prediction of response

## Abstract

**Background:**

Small cell lung cancer (SCLC) is an aggressive cancer often presenting in an advanced stage and prognosis is poor. Early response evaluation may have impact on the treatment strategy.

**Aim:**

We evaluated ^18^F-fluorothymidine-(FLT)-PET/diffusion-weighted-(DW)-MRI early after treatment start to describe biological changes during therapy, the potential of early response evaluation, and the added value of FLT-PET/DW-MRI.

**Methods:**

Patients with SCLC referred for standard chemotherapy were eligible. FLT-PET/DW-MRI of the chest and brain was acquired within 14 days after treatment start. FLT-PET/DW-MRI was compared with pretreatment FDG-PET/CT. Standardized uptake value (SUV), apparent diffusion coefficient (ADC), and functional tumor volumes were measured. FDG-SUV_peak_, FLT-SUV_peak_, and ADC_median_; spatial distribution of aggressive areas; and voxel-by-voxel analyses were evaluated to compare the biological information derived from the three functional imaging modalities. FDG-SUV_peak_, FLT-SUV_peak_, and ADC_median_ were also analyzed for ability to predict final treatment response.

**Results:**

Twelve patients with SCLC completed FLT-PET/MRI 1–9 days after treatment start. In nine patients, pretreatment FDG-PET/CT was available for comparison. A total of 16 T-sites and 12 N-sites were identified. No brain metastases were detected. FDG-SUV_peak_ was 2.0–22.7 in T-sites and 5.5–17.3 in N-sites. FLT-SUV_peak_ was 0.6–11.5 in T-sites and 1.2–2.4 in N-sites. ADC_median_ was 0.76–1.74 × 10^− 3^ mm^2^/s in T-sites and 0.88–2.09 × 10^−3^ mm^2^/s in N-sites. FLT-SUV_peak_ correlated with FDG-SUV_peak_, and voxel-by-voxel correlation was positive, though the hottest regions were dissimilarly distributed in FLT-PET compared to FDG-PET. FLT-SUV_peak_ was not correlated with ADC_median_, and voxel-by-voxel analyses and spatial distribution of aggressive areas varied with no systematic relation. LT-SUV_peak_ was significantly lower in responding lesions than non-responding lesions (mean FLT-SUV_peak_ in T-sites: 1.5 vs. 5.7; *p* = 0.007, mean FLT-SUV_peak_ in N-sites: 1.6 vs. 2.2; *p* = 0.013).

**Conclusions:**

FLT-PET and DW-MRI performed early after treatment start may add biological information in patients with SCLC. Proliferation early after treatment start measured by FLT-PET is a promising predictor for final treatment response that warrants further investigation.

**Trial registration:**

Clinicaltrials.gov, NCT02995902. Registered 11 December 2014 - Retrospectively registered.

## Introduction

Functional imaging, such as positron emission tomography (PET) and diffusion-weighted magnetic resonance imaging (DW-MRI), are important tools to gain non-invasive information about tumor biology and tumor heterogeneity. ^18^F-fluorodeoxy-glucose (FDG)-PET/CT has established its role in staging of small cell lung cancer (SCLC) (Ruben and Ball 2012) and causes stage migration in up to 40% of the patients influencing the choice of treatment and outcome (van Loon et al. 2011). FDG-PET has shown prognostic value in SCLC (Langer et al. 2014; Lee et al. 2014; Park et al. 2014; Aktan et al. 2017; Kim et al. 2018; Mirili et al. 2019; Fu et al. 2018; Chang et al. 2019), but the potential of FDG-PET for early response evaluation remains unclear (Yamamoto et al. 2009; Fischer et al. 2006).

SCLC is an aggressive cancer with more than two-thirds of the patients presenting in stage IV (Dayen et al. 2017). Over the last three decades, improvements for patients with SCLC have been sparse. However recently, new drug classes including immune check point inhibitors (Ready et al. 2018; Horn et al. 2018) and transcription inhibitors (Luis et al. 2019) have raised hope for improving the treatment results. Accordingly, the need for a better understanding of tumor biology and prognostication is higher than ever.

^18^F-fluorothymidine (FLT) is a PET-tracer of proliferation (Yap et al. 2006; Brockenbrough et al. 2011). FLT-PET has been studied in SCLC xenografts in mice showing promise for early response evaluation of treatment with epidermal growth factor receptor tyrosine kinase inhibitors (EGFR TKI) (Pardo et al. 2009), but we are unaware of any studies of FLT-PET in patients with SCLC. In patients with non-small cell lung cancer (NSCLC), pretreatment FLT-PET/CT and FLT-PET/CT early after treatment start has shown prognostic value (Yap et al. 2006; Brockenbrough et al. 2011; Usuda et al. 2018; Kahraman et al. 2012; Trigonis et al. 2014). Early response evaluation measured by FLT-PET/CT was prognostic for progression-free survival (PFS) in patients with NSCLC treated with EGFR TKI (Kahraman et al. 2011; Mileshkin et al. 2011; Sohn et al. 2008). Results from patients treated with a platin-based chemotherapy (Crandall et al. 2017) and concurrent chemo-radiotherapy were, however, non-significant (Trigonis et al. 2014; Everitt et al. 2017). In contrary to FDG, FLT does not cross the blood-brain barrier if intact (Nikaki et al. 2017). FLT-PET is however able to detect brain tumors (Nikaki et al. 2017) and brain metastases (Nguyen et al. 2018; Dittmann et al. 2003; Nakajo et al. 2013; Hoshikawa et al. 2013) possible due to disruption of the blood brain-barrier in these patients.

DW-MRI measures water diffusion within the tissue which is affected by micro textural features. Tumors with a high cell density and a poor differentiation have restricted water diffusion, which can be quantified by a lower apparent diffusion coefficient (ADC) (Weiss et al. 2016). A meta-analysis has shown that ADC can distinguish malignant lesions in the lungs from benign lesions and that ADC is lower in SCLC than NSCLC (Shen et al. 2016). ADC change after therapy has proven prognostic of overall survival (OS) in a study mixed of patients with SCLC and NSCLC (Tsuchida et al. 2013). Other studies of patients with NSCLC have confirmed predictive and prognostic value of ADC change during therapy (Weiss et al. 2016; Yabuuchi et al. 2011; Yu et al. 2014), though baseline ADC did not show prognostic value (Usuda et al. 2018; Yu et al. 2014).

The objectives of this study were to pilot the potential of FLT-PET and DWI-MRI early after treatment start in patients with SCLC; for early evaluation of tumor biology during treatment and for early response evaluation.

To our knowledge, this is the first study to examine FLT-PET in patients with SCLC.

## Methods

### Patients

Patients with histologically verified SCLC, referred to first line standard chemotherapy, and patients with relapsed SCLC referred to reinduction of standard chemotherapy, were eligible. Patients were recruited at the Dept. of Oncology, Rigshospitalet, Denmark from November 2014 to May 2017. All patients gave informed consent, and the study was approved by the local ethics committee, approval number H-1-2014-026.

### Imaging

FLT-PET/MRI was performed within 14 days after start of chemotherapy on an integrated PET/MRI system (Siemens Biograph mMR) with a 3-T magnet. FLT (5 MBq/kg, max 350 MBq) was injected 60 min prior to PET/MRI, without restrictions regarding fasting or resting. PET and MRI were conducted simultaneously as static, regional images starting with one bed position over cerebrum followed by one bed position over thorax centered on the primary tumor. T1-weighted imaging with and without gadolinium contrast, T2-weighted imaging, and DWI were acquired over both bed positions using the following protocol:

Cerebrum: 3D VIBE for PET attenuation correction (echo time (TE) 4.00 ms; repetition time (TR) 8.6 ms; voxel size 1.1 × 1.0 × 7.0 mm^3^); sagittal T1 MPRAGE (TE 2.44 ms; TR 1900 ms; voxel size 1.0 × 1.0 × 1.0 mm^3^); transverse T2 BLADE (TE 117 ms; TR 5550 ms; voxel size 0.7 × 0.7 × 5.0 mm^3^); DWI using single-shot echo-planar imaging (EPI) (TE 101 ms; TR 6800 ms; voxel size 1.1 × 1.1 × 4.0 mm^3^, *b* values of 0 and 800 s/mm^2^). Thorax: 3D VIBE for PET attenuation correction (TE 1.23/2.46 ms; TR 3.60 ms; voxel size 4.1 × 2.6 × 3.1 mm^3^); transverse T1 VIBE in breath-hold (TE 1.23 ms; TR 3.46 ms; voxel size 1.7 × 1.3 × 4.0 mm^3^); DWI using single-shot EPI triggered to the position of the diaphragm (TE 73 ms; TR 2200 ms; voxel size 3.7 × 3.0 × 5.0 mm^3^; *b* values of 0, 150, 400, and 800 s/mm^2^); coronal T2 BLADE in four breath-holds with Gadolinium-based contrast (TE 138 ms; TR 2030 ms; voxel size 1.4 × 1.4 × 6.0 mm^3^); transverse T1 VIBE in breath-hold employing a small shim volume covering the tumor, with Gadolinium-based contrast (TE 1.23 ms; TR 3.46 ms; voxel size 1.7 × 1.3 × 4.0 mm^3^). Cerebrum: sagittal T1 MPRAGE with Gadolinium-based contrast (TE 2.44 ms; TR 1900 ms; voxel size 1.0 × 1.0 × 1.0 mm^3^).

FLT-PET data were reconstructed using ordinary Poisson 3D ordered subset expectation maximization (OP-OSEM) with three iterations, 21 subsets, voxel size 2.1 × 2.1 × 2.0 mm^3^ with Siemens standard MR-based Dixon attenuation correction, and 4 mm post-filtering.

If pretreatment FDG-PET/CT had been performed, this was included in the study. Pretreatment FDG-PET/CT was at different hospitals by clinical indication. Accordingly, pretreatment FDG-PET/CT was performed on different scanner models and variant PET-protocols (details available in Additional file 1: Table S1).

### Image analysis

All imaging datasets were analyzed on a Mirada Medical Ltd. XD 3.6 workstation (MIRADA Medical, Oxford, UK).

Metabolic tumor volume (MTV) was delineated on pretreatment FDG-PETs by thresholds of 41% and 50% of maximum standardized uptake value (SUV_max_) (MTV41 and MTV50), as recommended by the European Association of Nuclear Medicine (EANM) procedure guidelines (Boellaard et al. 2015).

Proliferative tumor volume (PTV) being the functional tumor volume by FLT-PET (equivalent to MTV by FDG-PET) was delineated on posttreatment FLT-PETs using the same thresholds as recommended for FDG-PET (PTV41 and PTV50) as well as with an absolute threshold of SUV = 1.4 (PTV1.4), as recommended by Thureau et al. (2013). Within the above tumor volumes, volume, SUV_max_, SUV_peak_, and SUV_mean_ were measured. Total lesion glycolysis (TLG) by FDG-PET and total lesion proliferation (TLP) by FLT-PET were calculated by multiplying MTV and PTV with the corresponding SUV_mean_ (e.g., TLG41 = MTV41 × SUV_mean_41; TLP50 = PTV50 x SUV_mean_50) for each tumor volume.

Diffusion-weighted tumor volume (DWTV25) was delineated on DW-MRIs (*b* = 800 s/mm^2^) using a threshold of 25% of maximum. DWTV25 was projected to the ADC-map for quantifying ADC_mean_ and ADC_median_.

In addition, volumes of the primary tumor, lymph nodes, and distant metastases included in the MRI field of view were contoured by an experienced radiologist on T1-weighted MRI with gadolinium contrast, following recommendations for delineation of gross tumor volume (GTV) (Nestle et al. 2018). The MRI contours were projected to FDG-PET, FLT-PET, and the ADC-map for voxel-by-voxel analyses comparing the modalities. Image modalities were rigidly registered and subsequently resampled to identical voxel sizes. Voxel-by-voxel analysis was considered reasonable when the measured position of lesional landmarks in registered scans deviated by less than 10 mm in the direction of maximum displacement *and* if visual inspection indicated good overall alignment, or in lesions with no characteristic landmarks, good overall alignment by visual inspection. If visual inspection indicated adequate overall alignment, a maximum of 5 mm in the direction of maximum displacement was considered reasonable for voxel-by-voxel analysis.

Within each lesion, we spatially compared the most “aggressive” areas within the tumor defined by each scan modality. The most aggressive areas were defined as the area with highest metabolism or highest proliferation measured by FDG-PET and FLT-PET, respectively, and by DW-MRI as the areas with lowest diffusion. The most aggressive areas were delineated on FDG-PET and FLT-PET using a threshold of 70% of SUV_max_ (MTV70; PTV70), and on DW-MRI (*b* = 800 s/mm^2^) using a threshold of 50% (DWTV50).

Overlap of MTV70 vs. PTV70 and PTV70 vs. DWTV50 were analyzed visually. Overlap were graded as no overlap, partial overlap (< 50% overlap), or high overlap (> 50% overlap).

As a quality control, FLT uptake within normal tissue was measured, as recommended by Cysouw et al. (2017). Briefly, liver FLT-uptake was measured in a 3-cm-diameter sphere placed in the right upper lobe of the liver; bone marrow FLT-uptake was measured in a 1-cm-diameter sphere placed in a lower thoracic vertebra; and FLT-uptake in the mediastinal blood pool was measured in a cylinder of 1 × 2 cm in ascending aorta. Metastases, previously irradiated tissue, and the aortic wall were avoided. Within these volumes, FLT-SUV_max_, FLT-SUV_peak_, and FLT-SUV_mean_ were measured.

### Follow up and outcome

Patients were followed until 1 year after the last patient had completed FLT-PET/MRI.

Final response to treatment was determined by routine CT using The Response Evaluation Criteria in Solid Tumors (RECIST) 1.1 (Eisenhauer et al. 2009).

Response of each lesion was defined by the same limits as used in RECIST 1.1 (response: > 30% decrease of longest lesion diameter; progression: > 20% increase of longest lesion diameter; no change: neither response nor progression).

PFS was defined as time from PET/MRI to progression or death of any cause, whichever occurred first. OS was defined as time from PET/MRI to death of any cause.

### Statistics

Statistical analyses were performed in SPSS, version 25.

Correlation analyses of PET-parameters and MRI-parameters across patients were performed using Spearman’s rank correlation. Within-patient voxel-by-voxel analyses were performed by linear regression.

Differences of each PET- and MRI-parameter in lesions with response vs. lesions with no change or progression were tested by an independent *t* test for response prediction. Levene’s test was used for test of equality of variances and if variances were not equal; data was transformed by the natural logarithm prior to the independent *t* test analysis. PFS and OS were calculated using the Kaplan-Meier method. *P* < 0.05 was considered statistically significant.

## Results

### Patient data

**Fig. 1:**
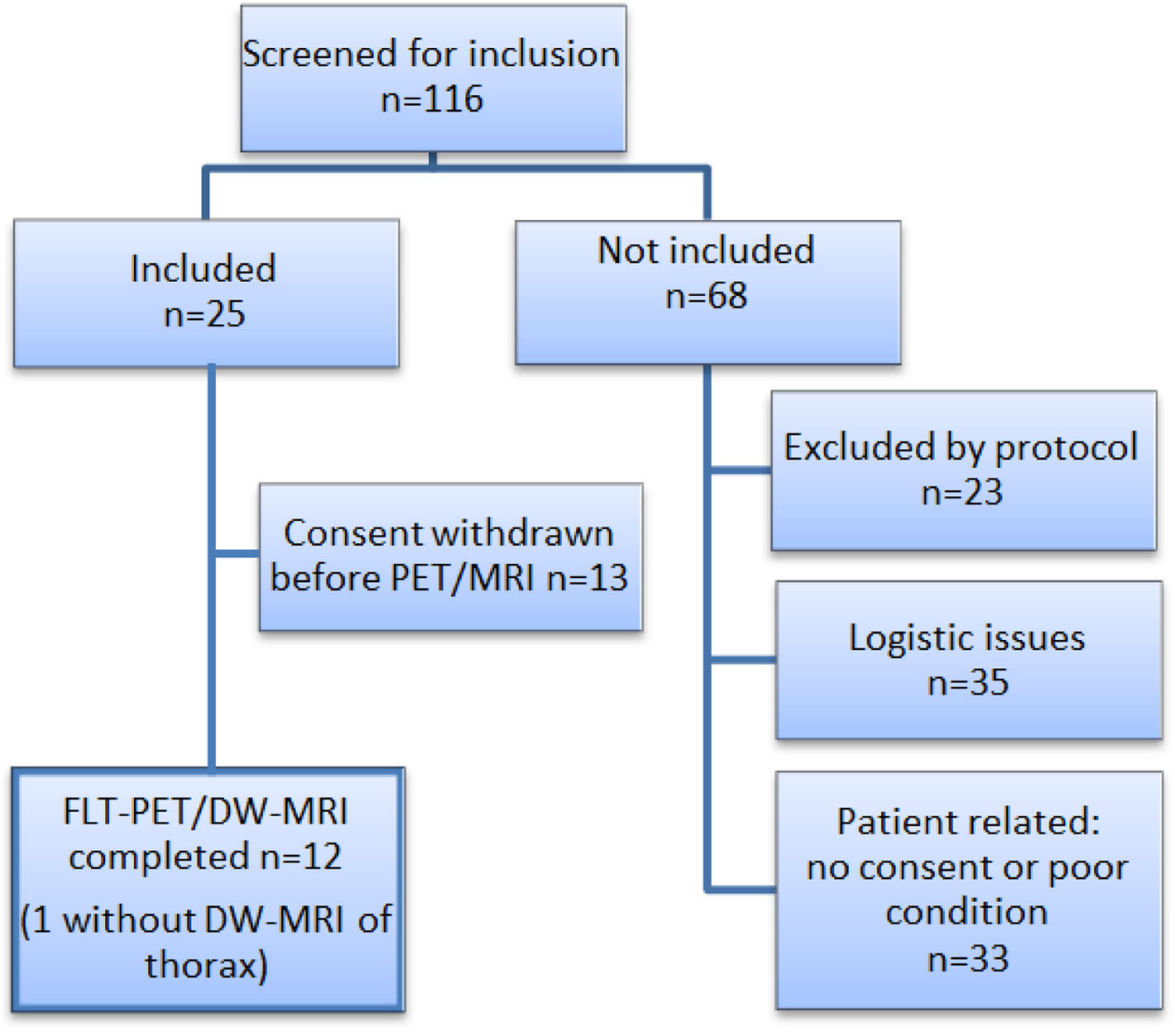
Overview of the inclusion process

FLT-PET/MRI was conducted in 12 patients, but in one patient, DW-MRI of thorax failed. Figure 1 provides an overview of the inclusion process. Table 1 presents the characteristics and outcome of the 12 patients. Nine patients had extensive disease (ED); one patient had limited disease (LD); and two patients had a relapse of SCLC and had previously been treated with concomitant chemo-radiotherapy. All patients were either active or former smokers with 40–60 pack-years.

**Table 1:** Patient characteristics and outcome

Pt no.	Age	Sex	VALSG stage	PS	LDH	Treatment	Best response	PFS (days)	OS (days)	Follow up (days)
1	62	f	ED	1	220	Car+eto × 6	CR	246	349	
2	60	f	ED	1	231	Car+eto × 6 RT 30 Gy(bone metastases) PCI	PR	194	320	
3	72	m	ED	1	216	Car+eto × 6	PR	134	209	
4	77	m	ED	1	354	Car+eto × 3	SD	90	114	
5	59	f	ED/Relapse	2	267	Car+eto × 3	SD	220	220	
6	58	m	LD	0	172	Cis + eto × 2, car+eto × 2 Concomitant RT 60 Gy (RUL + mediastinum) PCI	CR	155		741
7	76	m	ED	3	1180	Eto × 1 RT (bone metastases)	NA*	47	47	
8	51	f	ED	1	160	Car+eto × 6 Sequential RT 30 Gy (mediastinum) PCI	PR	243		460
9	60	f	ED	1	264	Car+eto × 6	PR	195	321	
10	70	f	ED/Relapse	1	173	Car+eto × 3	PR	124	347	
11	59	m	ED	0	736	Car+eto × 6 Sequential RT 30 Gy (Right lung + mediastinum) RT 30 Gy (metastases on the thoracic wall and on scull)	SD	275	478	
12	74	m	ED	0	NA	Car+eto × 4 Sequential RT 30 Gy (mediastinum, neck)	PD	50	95	

*f* female, *m* male, *VALSG stage* The Veteran’s administration Lung Study Group two stage classification scheme, *ED* extensive disease, *LD* limited disease, *PS* WHO performance status, *LDH* blood lactate dehydrogenase, *NA* not available, *car* carboplatin, *eto* etoposide, *RT* radiotherapy, *PCI* prophylactic cranial irradiation, *cis* cisplatin, *RUL* right upper lobe, *CR* complete response, *PR* partial response, *SD* stable disease, *PD* progressive disease, *PFS* progression free survival, *OS* overall survival; * No response evaluation, as the patient died prior to evaluation

All patients were treated with chemotherapy; 11 patients received at least three cycles of cis- or carboplatin and etoposide; one patient received only one cycle of etoposide. The patient with LD received concomitant radiotherapy (RT) to 45 Gy, and three patients with ED received sequential RT to 30 Gy; in two patients due to poor response to chemotherapy, and in one patient as consolidation treatment.

Median OS was 10.5 months, and median PFS was 5.1 months. Evaluated on CT, two patients had complete response, five patients had partial response, three patients had stable disease, one patient had progressive disease, and one patient had no relevant follow up scans as he died 1.5 months after treatment start. The seven responders all had relapse within 4 months after last exposure to chemotherapy.

### Scan data

FLT-PET/MR was performed 1–9 days after start of chemotherapy (median 4.5 days). Pretreatment FDG-PET/CT was available in nine patients. FDG-PET/CT was performed 7–21 days before FLT-PET/DW-MRI (median 16 days). An overview of scan data and times is presented in Additional file 1: Table S1.

### Malignant lesions

A total of 32 lesions were analyzed; 16 T-sites, 12 N-sites, and 4 M-sites. T-, N-, and M-sites will be described separately.

Table 2 provides an overview of identified lesions, selected parameters, and lesion-specific outcome (all PET- and MRI-parameters are available in Additional file 2: Table S2). In many lesions, FLT-uptake was low compared with background uptake, and, accordingly, delineation of PTV was not possible in three of 16 T-sites and nine of 12 N-sites. Sufficient alignment was not achieved in all lesions and voxel-by-voxel analysis for FDG-PET vs. FLT-PET was feasible in only four T-sites, seven N-sites, and two M-sites. Voxel-by-voxel analyses for FLT-PET vs. DW-MRI were feasible in nine T-sites, nine N-sites, and two M-sites. The alignment of FDG-PET and DW-MRI was generally poor, and no voxel-by-voxel analyses of FDG-PET vs. ADC were feasible.

**Table 2:** Malignant lesions: location, FDG-SUV_peak_, FLT-SUV_peak_, ADC_median_, and outcome

Pt no	Lesion no	Location	FDG-PET	FLT-PET	DW-MRI	Lesion outcome		Comments
			SUV_peak_	SUV_peak_	ADC_median_	Lesion response	Progression (days)	
1	1-T	LLL	16.5	1.6	1.22	Response	–	
	1-N1	2R + 4R	12.2	1.8	1.21	Response	–	
	1-N2	10L + 11L	13.4	1.5	0.90	Response	–	
	1-N3	4L	15.5	1.9	0.88	Response	–	
	1-N4	7	17.3	2.1	1.07	Response		
	1-M	RUL	4.2	1.1	1.05	Response	–	
2	2-N	2R + 4R + 4L + 7	NA	1.3	1.54	Response	194	
3	3-T	LLL + hilus sin	9.0	2.1	1.59	Response	–	
	3-N1	8	8.1	1.6	1.96	Response	–	
	3-N2	7	5.5	1.2	1.89	Response	–	
	3-N3	4L + 4R + 5	8.0	1.3	1.39	Response	134	
4	4-T	LUL	22.7	11.5	1.43	No change	90	
5	5-T	Hilus dxt	NA	2.6	1.74	No change	–	Previously irradiated
6	6-T	RLL	3.9	0.6	#	Response	155	
	6-N	10-11R	6.2	1.3	2.09	Response	–	
7	7-T	LUL + hilus + med	8.3	4.0	NA	NA	NA	No outcome evaluation as the patient died day 47
	7-M	Lymph node in left axilla	5.2	1.9	NA	NA	NA	No outcome evaluation as the patient died day 47
8	8-T1	RUL + med	9.7	1.7	1.11	Response	–	
	8-T2	RUL	2.0	0.6	#	Response	–	
	8-T3	RUL	2.2	0.7	#	Response	–	
9	9-T1	LUL + med	12.1	1.7	1.74	Response	195	
	9-T2	LUL	3.7	1.3	1.01	Response	–	
10	10-T1	hilus sin + med	NA	2.8	0.82	Response	124	Previously irradiated
	10-T2	lingula	NA	1.9	1.10	Response	–	
11	11-T1	RUL + med	11.8	3.0	1.15	No change	275	
	11-T2	RUL	4.4	1.0	0.76	NA	–	Response evaluation not possible due to atelectasis
	11-M1	Subcutis + os frontale	8.9	2.3	1.03	No change	–	
	11-M2	Subcutis + costa	10.9	4.6	1.03	No change	–	
12	12-T	RUL	12.8	1.2	1.19	Response	–	
	12-N1	4 + 7	11.6	2.4	1.56	Progression	50	
	12-N2	7 + 8	10.2	2.2	1.85	Progression	50	
	12-N3	10-11R	13.7	1.9	1.61	Progression	50	

*LLL* left lower lobe, *RUL* right upper lobe, *2R, 4R, etc* lymph node stations, *LUL* left upper lobe, *RLL* right lower lobe; med: mediastinum, *FDG* ^18^F-fluorodeoxy-glucose, *PET* positron emission tomography, *SUV* standardized uptake value, *FLT* ^18^F-fluorothymidine, *DW* diffusion weighted, *MRI* magnetic resonance imaging, *ADC* apparent diffusion coefficient, *NA* not available, # Tumor not visible on MRI and/or DW-MRI

All SUVs from FDG-PET (FDG-SUV_max_, FDG-SUV_peak_, FDG-SUV_mean_41, and FDG-SUV_mean_50) were significantly correlated (*p* < 0.001), as were all SUVs from FLT-PET (FLT-SUV_max_, FLT-SUV_peak_, FLT-SUV_mean_41, FLT-SUV_mean_50, and FLT-SUV_mean_1.4), all ADCs (ADC_mean_ and ADC_median_), and all tumor volumes (MTV41, MTV50, PTV41, PTV50, PTV1.4, DWTV25, and GTV). Therefore, for further evaluation, only FDG-SUV_peak_, FLT-SUV_peak_, ADC_median_, MTV41, PTV50, DWTV25, TLG41, and TLP50 are presented.

### T-sites

From the nine patients who had a pretreatment FDG-PET/CT, all T-sites (*n* = 13) were detectable by FDG-PET. FDG-SUV_peak_ varied from 2.0 to 22.7.

All patients completed FLT-PET, but from the 16 T-sites, only five T-sites had an FLT-uptake clearly distinguishable from background. These five T-sites had a heterogeneous FLT-uptake and only a fraction of the tumor had a highly visible FLT-uptake. FLT-SUV_peak_ from the 16 T-sites varied from 0.6 to 11.5.

From the 11 patients whom completed DW-MRI, 12 of 15 T-sites were detectable by DW-MRI, and ADC_median_ varied from 0.76 to 1.74 × 10^−3^ mm^2^/s. Another three T-sites had no signal on DW-MRI: on pretreatment FDG-PET/CT, these were all small with a diameter of maximum 1.6 cm.

**Fig. 2:**
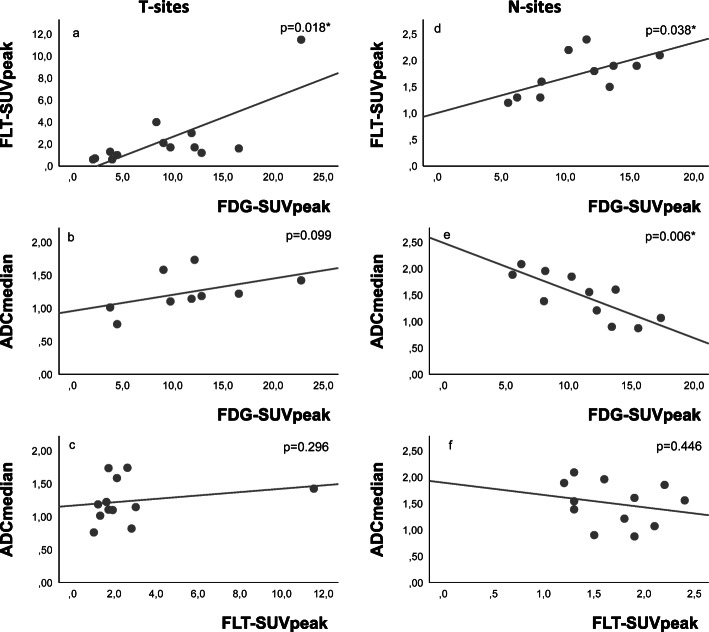
Correlations of FDG-SUV_peak_, FLT-SUV_peak_, and ADC_median_ in T-sites (**a**–**c**) and N-sites (**d**, **e**). FDG-SUV_peak_ vs. FLT-SUV_peak_ were positive correlated in T-sites (**a**) and N-sites (**d**). FDG-SUV_peak_ vs. ADC_median_ was not significantly correlated in T-sites (**b**), but significantly negative correlated in N-sites (**e**). ADC_median_ vs. FLT-SUV_peak_ were neither correlated in T-sites (**c**) nor N-sites (**f**). Significant correlations (*p* < 0.05) are marked with *

In each lesion, FLT-SUV_peak_ was lower than FDG-SUV_peak_. FDG-SUV_peak_ and FLT-SUV_peak_ were significantly correlated (*p* = 0.018), as shown in Fig. 2a. ADC_median_ was not significantly correlated with FDG-SUV_peak_ or FLT-SUV_peak_, as shown in Fig. 2b, c.

Due to low FLT-uptake, limited spatial information was available in many lesions. Accordingly, comparison of the most “aggressive” regions on FDG-PET and FLT-PET were possible only in three T-sites. Within these three T-sites, the “aggressive” regions were distributed unevenly and there was no overlap of MTV70 and PTV70.

Voxel-by-voxel analysis comparing FDG-PET vs. FLT-PET was feasible in four T-sites. In three of four lesions, the overall voxel-by-voxel correlation of FDG-PET and FLT-PET was moderate (*r* = 0.49–0.50), but the voxels with highest FLT-uptake were randomly distributed along the FDG-uptake-scale, confirming that the overall correlation is not applicable for the hottest voxels. The fourth voxel-by-voxel analysis showed a weak correlation.

Comparison of the most “aggressive” regions of FLT-PET and DW-MRI was possible in four T-sites: Two T-sites had a partial overlap of PTV70 and DWTV50, and two T-sites had no overlap of PTV70 and DWTV50. There was no systematic correlation on the voxel-by-voxel analysis of FLT-PET and ADC (*r* = − 0.66 to 0.42, *n* = 9).

**Fig. 3:**
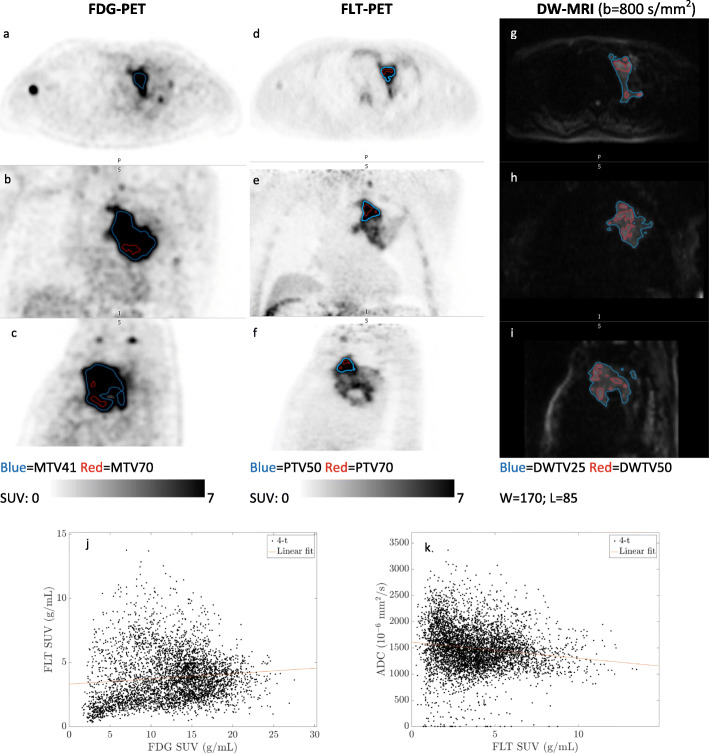
T-site (4-T) with high and heterogeneous FLT-uptake: FDG-PET (axial (**a**), coronal (**b**), sagittal (**c**)), FLT-PET (axial (**d**), coronal (**e**), sagittal (**f**)), and DW-MRI (transversal (**g**), coronal (**h**), sagittal (**i**)), and voxel-by-voxel scatterplot of FDG-SUV vs. FLT-SUV (**j**) and FLT-SUV vs. ADC (**k**). This lesion was clearly detectable on FDG-PET (SUV_peak_ 22.7), detectable but very heterogeneous on FLT-PET (SUV_peak_ 11.5), and detectable on DW-MRI (ADC_median_ 1.43 × 10^−3^ mm^2^/s). The most metabolically active region (MTV70) was located caudally, whereas the most proliferative active region (PTV70) was located cranially within the tumor, thus MTV70 and PTV70 showed no overlap. The most water-diffusion restricted regions (DWTV50) were randomly distributed and overlapped partially with both MTV70 and PTV70. The voxel-by-voxel scatter plots (**j**, **k**) showed very weak overall correlations. This T-site had no change as response to chemotherapy

**Fig. 4:**
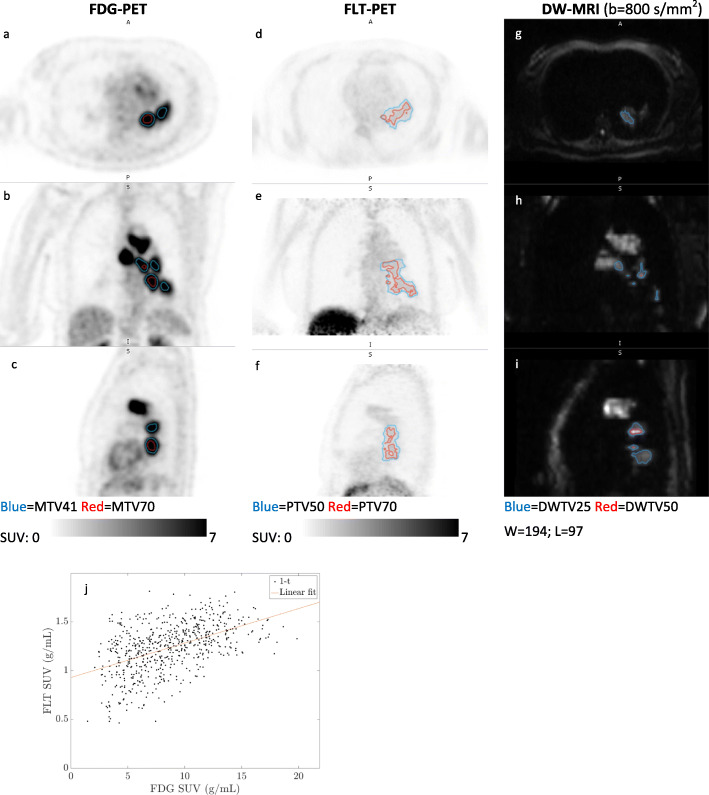
T-site (1-T) with low FLT-uptake: FDG-PET (axial (**a**), coronal (**b**), sagittal (**c**)), FLT-PET (axial (**d**), coronal (**e**), sagittal (**f**)), and DW-MRI (transversal (**g**), coronal (**h**), sagittal (**i**)), and voxel-by-voxel scatterplot of FDG-SUV vs. FLT-SUV (**j**). This T-site was clearly detectable on FDG-PET (SUV_peak_ 16.6); almost indistinguishable from background on FLT-PET (SUV_peak_ 1.6); and detectable on DW-MRI (ADC_median_ 1.22 × 10^−3^ mm^2^/s). The low tumor-to-background ratio causes PTV70 to be less convincing, visually. MTV70 and DWTV50 had a partial overlap. The voxel-by-voxel scatter plot of FLT-SUV and FDG-SUV (**j**) showed an overall moderate positive correlation (*r* = 0.50), but the very low FLT-SUVs should be noticed, and the correlation may be a result of perfusion to the region rather than a correlation between metabolism and (a very low) proliferation. Voxel-by-voxel analysis was not feasible for FLT-SUV vs. ADC. This T-site had complete response to chemotherapy, and did not relapse during follow up

Figures 3 and 4 are examples of two representative T-sites with high, respectively, low FLT-uptake. As illustrated by Figs. 3 and 4, the three imaging modalities show apparently different patterns of intra-tumor heterogeneity.

### N-sites

The 12 N-sites each consisted of a single lymph node or larger lymph node conglomerates, and therefore varied substantially in size (GTV: 3.9–119.7 cm^3^).

FDG-SUV_peak_ ranged from 5.5 to 17.3. FLT-uptake was distinguishable from background uptake only in three of 12 N-sites, all in the same patient. FLT-SUV_peak_ ranged from 1.2 to 2.4. ADC_median_ ranged from 0.88 to 2.09 × 10^−3^ mm^2^/s.

In each N-site, FLT-SUV_peak_ was lower than FDG-SUV_peak_, and their correlation was significant (*p* = 0.038), see Fig. 2d. ADC_median_ correlated negative with FDG-SUV_peak_ (*p* = 0.006), but there was no significant correlation between ADC_median_ and FLT-SUV_peak_, as illustrated in Fig. 2e, f.

Spatial comparisons were possible only in the three N-sites, due to the low detection rate by FLT-PET.

MTV70 and PTV70 showed partial or high overlap in all three N-sites, and voxel-by-voxel correlations of FDG-PET and FLT-PET was moderate and positive (*r* = 0.41–0.60). PTV70 and DWTV50 showed partial or high overlap, and voxel-by-voxel correlations of FLT-PET and ADC were weak and negative (*r* = − 0.44 to − 0.15).

**Fig. 5:**
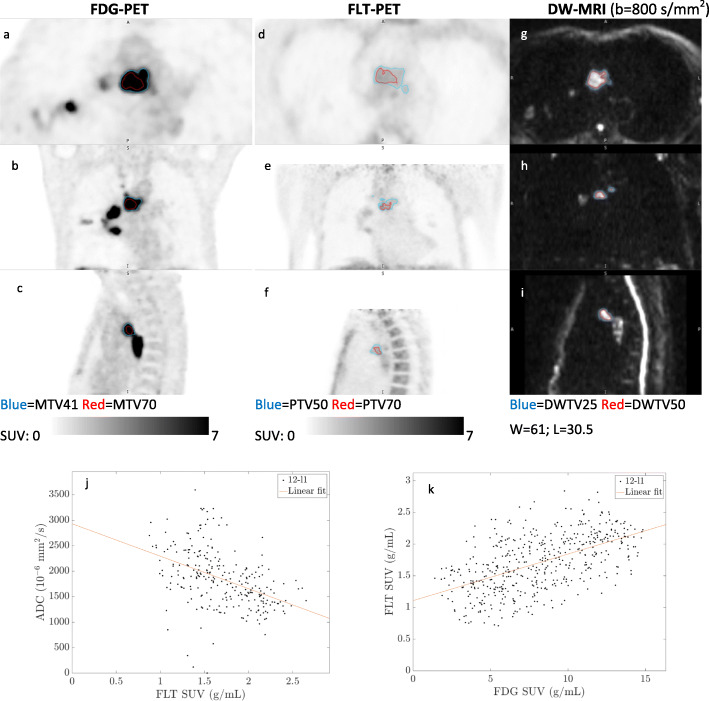
N-site (12-N1): FDG-PET (axial (**a**), coronal (**b**), sagittal (**c**)), FLT-PET (axial (**d**), coronal (**e**), sagittal (**f**)), and DW-MRI (transversal (**g**), coronal (**h**), sagittal (**i**)), and voxel-by-voxel scatterplot of FDG-SUV vs. FLT-SUV (**j**), and FLT-SUV vs. ADC (**k**). This N-site were clearly detectable on FDG-PET (SUV_peak_ 11.6), FLT-uptake was the highest of all N-sites (SUV_peak_ 2.4), and detectable on DW-MRI (ADC_median_ 1.56 × 10^−3^ mm^2^/s). The most aggressive regions (MTV70, PTV70, and DWTV50) were located centrally within the tumor on all imaging modalities, and voxel-by-voxel correlations were moderate for FDG-SUV vs. FLT-SUV (*r* = 0.55) (**j**) and for FLT-SUV vs. ADC (*r* = − 0.44) (**k**). This N-site progressed during chemotherapy

Figure 5 illustrates an N-site that could be visualized by all three imaging modalities. As illustrated with this N-site, but applicable for all three N-sites that were detectable by all three imaging modalities, the most “aggressive” regions showed partial or high overlap, but most lesions were undetectable by FLT-PET.

### M-sites

No brain metastases were detected by FDG-PET, FLT-PET, DW-MRI, or MRI. M-sites were detected in the lung, in an axillary lymph node, two in subcutis, two in bones (vertebras), and several in the liver. Parameters from the four metastases in the lung, axilla, and subcutis are available in Table 2, but due to the small number and the heterogeneity of localization, no further analyses were conducted.

### Prediction of final response to treatment

Of the 28 T- and N-sites, 20 responded to chemotherapy: three T-sites had no change and three N-sites progressed during chemotherapy. Another two lesions were not response evaluated; one because the patients died prior to evaluation (7-T); and one because it was incorporated in atelectasis and not evaluable after 6 cycles (11-T2).

No T-sites progressed during chemotherapy, and no N-sites had no change, therefore comparing analyses were performed of T-sites with response vs. no change, and N-sites with response vs. progression.

MTV41, TLG41, FLT-SUV_peak_, and TLP50 were significantly lower in T-sites with response than T-sites with no change (mean MTV41: 41 vs. 208 cm^3^; *p* = 0.002; mean TLG41: 311 vs. 2410; *p* = 0.006; mean FLT-SUV_peak_: 1.5 vs. 5.7; *p* = 0.007; mean TLP50: 35.5 vs. 120.5; *p* = 0.029). In N-sites, FLT-SUV_peak_ was significantly lower in responding N-sites than N-sites with progression (mean FLT-SUV_peak_ 1.6 vs. 2.2; *p* = 0.013).

FDG-SUV_peak_, TLG41, PTV50, ADC_median_, and DWTV did not show any difference in responding vs. no change T-sites, or progressing N-sites, neither did MTV41 and TLP50 from N-sites.

**Fig. 6:**
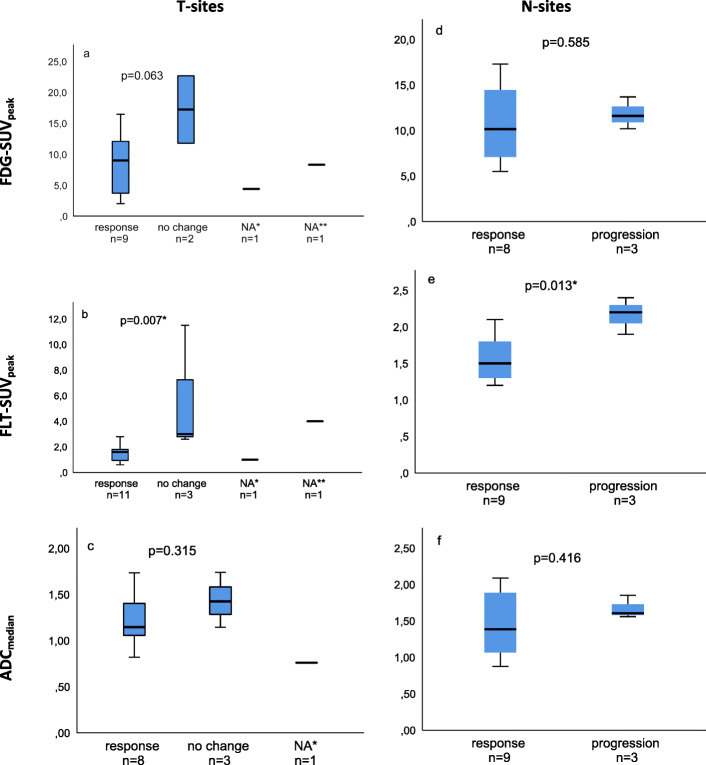
FDG-SUV_peak_, FLT-SUV_peak_, and ADC_median_ in lesions with response vs. lesions with no change or progression. T-sites are shown in left panel (**a**–**c**) and N-sites in right panel (**d**–**f**). Note that no T-sites progressed during chemotherapy, and no N-sites had no change. FLT-SUV_peak_ was significantly different in T-sites with response vs. no change (**b**) and in N-sites with response vs. progression (**e**). Three lesions with response had no signal on DW-MRI and are not included (**c**). Lesions that were not evaluated for response are included for completeness. NA*: response evaluation was not available due to atelectasis. NA**: The patient died prior to any response evaluation

The differences of FDG-SUV_peak_, FLT-SUV_peak_, and ADC_median_ in responding vs. no change or progressive lesions are illustrated in Fig. 6. As seen in Fig. 6b, FLT-SUV_peak_ was lower in all, but one responding T-site (FLT-SUV_peak_ 0.6–2.8) compared with T-sites with no change (FLT-SUV_peak_ 2.6–11.5). All comparing analyses are available in Additional file 3: Table S3.

### FLT uptake in normal tissue

FLT-uptake in normal tissue showed large variation across the patients. Liver FLT-SUV_peak_ ranged from 2.0 to 11.3 (reference: 3.46–7.46); blood pool FLT-SUV_peak_ ranged from 0.6 to 1.3 (reference: 0.44–1.04); and bone marrow FLT-SUV_peak_ ranged from 1.2 to 11.3 (reference: 4.86–11.36), reference values from (Cysouw et al. 2017). There were no significant correlations between normal tissue FLT-uptake and time from treatment start to FLT-PET/MRI or FLT-uptake time. FLT-SUV in the liver, blood pool, and bone marrow are available in Additional file 4: Table S4. In most cases, FLT-uptake in normal tissue was not within Cysouw’s reference interval (Cysouw et al. 2017). In particular, bone marrow FLT-uptake was lower than the reference interval in ten of 12 patients. Three patients had a lower liver FLT-uptake compared with the reference interval, and two patients had higher liver and blood pool FLT-uptake compared with the reference interval.

## Discussion

The aim of this study was to perform a pilot study of the potential of FLT-PET and DW-MRI early after treatment start in patients with small cell lung cancer. Our study indicates that FLT-PET and DW-MRI after one cycle of chemotherapy has a potential to add biological information to pretreatment FDG-PET, as the most proliferative active regions measured by FLT-PET, the most water diffusion restricted regions measured by DW-MRI and the most metabolically active regions measured by FDG-PET were all dissimilarly distributed.

We showed that persistent proliferation measured by FLT-PET 1–9 days after start of chemotherapy is a potential predictor of non-response to treatment, whereas the value of DW-MRI early after treatment start was unconvincing as ADC was not associated with final response.

The secondary aim of our study was to examine the added value of FLT-PET/DW-MRI in detection of brain metastases from SCLC. Unfortunately, we were not able clarify this issue, as none of the included patients had brain metastases.

It has previously been reported that up to 40% of patients with ED SCLC do not achieve objective response to first line therapy (Lattuca-Truc et al. 2019); therefore, early response evaluation to identify non-responders may have great impact. CT-response after the first cycle of chemotherapy in patients with LD SCLC has shown prognostic value of PFS and OS (Halvorsen TO et al. 2016; Fujii et al. 2012; Lee et al. 2015), but whether early CT-response can predict final treatment response has not been addressed, and patients with ED were not included in these studies (Halvorsen TO et al. 2016; Fujii et al. 2012; Lee et al. 2015). FDG-PET/CT has shown potential of early response evaluation in two studies (Yamamoto et al. 2009; Fischer et al. 2006), but each study identified only one non-responder; therefore, the ability to discriminated between responders and non-responders were less powerful. In the present study, we showed that FLT-PET early after treatment start has a potential to predict final response. A cut-off was not established, but the overlap of FLT-SUV_peak_ in responding vs. non-responding lesions was small.

We did not find any potential value of DW-MRI early after treatment start in patients with SCLC. DW-MRI has only been investigated sparsely in patients with SCLC after treatment start. Tsuchida et al. (2013) included 11 patients with SCLC in a study of a mixed lung cancer cohort: ADC after treatment in patients with SCLC was similar to our results: 0.91–1.97 × 10^− 3^ mm^2^/s, and absolute ADC after treatment was not associated with final response or OS. The change of ADC from baseline to early after treatment has shown predictive and prognostic value in patients with NSCLC (Weiss et al. 2016; Tsuchida et al. 2013; Yabuuchi et al. 2011; Yu et al. 2014). It seems ADC early after treatment start is less valuable than an ADC-change from baseline. The voxel-by-voxel correlations of FDG-PET, FLT-PET, and ADC were overall weak. Uncertainties of the intermodal image registration and varying respiration management strategies could potentially influence the voxel-by-voxel analysis. However, in consistence with the spatiovisual analysis, the results of the voxel-by-voxel analysis showed a dissimilar and heterogeneous distribution of the most aggressive regions of the modalities.

Recruiting patients to this study proved difficult and many potentially eligible patients were not included due to poor patient condition. To investigate the risk of a selection bias, we compared blood lactate dehydrogenase (LDH) and WHO performance status (PS) from a cohort of eligible, but not-included patients who attended our institution during the recruiting period and found no significant differences of LDH or PS (LDH: *p* = 0.663; PS: *p* = 0.053). Comparing our cohort with a recently published large French retrospective study of patients with SCLC from 1997 to 2017 (Lattuca-Truc et al. 2019), patients in our study had a better PS (PS ≥ 2: 17% vs. 44%), but more often ED (92% vs. 58%), poorer response rate (63% vs. 73%), and slightly shorter OS (10.5 months vs. 12.2 months). A systematic bias in the recruiting process is therefore not obvious.

This study has several technical limitations. We included pretreatment FDG-PET/CTs conducted according to varying clinical protocols of several referring hospitals; accordingly, there were several technical variations from patient to patient, and the FDG-PET parameters should be interpreted with caution. FLT-PET/MRI was performed over cerebrum first and secondly over thorax, causing long FLT-uptake time before obtaining FLT-PET of thorax. MRI artifacts may affect the SUV quantification, as described in FDG-PET/MR (Olin et al. 2018). Reproducibility of FLT-SUV quantification after MRI attenuation correction has not been established, but for FDG-PET/MRI, the reproducibility is high (Rasmussen et al. 2015). As a FLT-PET quality control, we assessed FLT-uptake in normal tissue for comparison with previously suggested references (Cysouw et al. 2017). In many cases, normal tissue FLT-uptake in our patients was not comprised within the reference intervals. In particular, bone marrow FLT-uptake was lower in ten of 12 patients, but also blood pool and liver FLT-uptake deviated from the references. The numerous outliers could have biological and/or technical explanations. Firstly, the known issue of detection of bone in Dixon MR-based attenuation (MRAC) correction may affect the measured FLT-uptake in the bone marrow as it is surrounded by bone (Samarin et al. 2012; Keller et al. 2013). Secondly, the reference intervals were established from a FLT-PET with a FLT-uptake time of 60 min, whereas FLT-uptake time in our study was 69–84 min. Thirdly, the PET reconstruction variables such as choice of reconstruction method (e.g., w/o time of flight and resolution modeling), number of iterations, and subsets and variations on correction methods (scatter, randoms and attenuation correction in general) can also influence PET quantification significantly. Noise in low FLT-uptake regions such as the blood pool and bone marrow might also have considerable effect. Fourthly, Cysouw’s references was based on baseline imaging and based on patients treated with EGFR TKIs, and in this setting a slight increase in liver and bone marrow FLT-uptake after treatment was suggested. Our patients received a myelosuppresive treatment, and it has previously been shown that FLT-uptake in the bone marrow reflects the hematopoietic activity (Vercellino et al. 2017). In concordance with the lower bone marrow and liver FLT-uptake in our results, Leimgruber et al. found a decrease in liver and bone marrow FLT-uptake (median 31% and 22%, respectively) 2 weeks after treatment with cisplatin/etoposide in a concurrent radiotherapy regimen (Leimgruber et al. 2014). Fifthly, timing after treatment start may influence the effect of chemotherapy on normal tissue FLT-uptake. In our study, there was no significant correlation between the FLT-parameters and time from treatment start to FLT-PET/MR, but two patients in our study had FLT-PET/MRI conducted only 1 day after treatment start, and they both had higher FLT-uptake in the liver and in the blood pool than the remaining patients and higher than the references. It is plausible that different anticancer treatments affect proliferation in normal tissues differently, and the deviations of normal tissue FLT-uptake in this study from the references could solely originate from biologically induced changes. Despite the presence of technical limitations, we believe that the tendencies in this study are trustworthy.

With the recent introduction of new treatments, there is an urgent need for larger studies to determine the diagnostic accuracy and implication of early treatment response. Preclinical studies and studies of other cancers than SCLC have shown that FLT-SUV_max_ reduces more rapidly and/or more pronounced than FDG-SUV_max_ during therapy (Kahraman et al. 2012; Jensen et al. 2010; Mudd et al. 2012; Kishino et al. 2012), but individual treatments may affect FDG- and FLT-uptake changes differently (Jensen and Kjaer 2015), and thus should be investigated separately. In studies of NSCLC, esophagus cancer, and lymphoma, early response evaluated by FLT-PET predicted final response better than FDG-PET (Gerbaudo et al. 2018; Minamimoto et al. 2016; Everitt et al. 2014), but it is not clear whether response by FLT-PET has superior prognostic value to FDG-PET, as results have been inconsistent (Kahraman et al. 2012; Mileshkin et al. 2011; Everitt et al. 2017). FLT-PET early after treatment start is a promising predictor for final response, but at this point, it is not clear which imaging modality is most valuable. For further validating the value of FLT-PET in SCLC, including baseline FLT-PET and correlating FDG-PET and FLT-PET at the same phase of treatments, would be beneficial in future studies.

## Conclusions

Persistent proliferation measured by FLT-PET early after treatment start was associated with poor response to chemotherapy in patients with SCLC. Thus, FLT-PET is a potential tool for selecting patients to be considered for change of treatment. We found no association between DW-MRI early after treatment and the final response.

## Supplementary information

**Additional file 1: Table S1.** Scan data and time points

**Additional file 2: Table S2.** PET- and MRI-parameters from malignant lesions

**Additional file 3: Table S3.** Comparison of PET- and MRI-parameters in lesions with response vs. no change or progression.

**Additional file 4: Table S4.** FLT-uptake in normal tissue.

## Data Availability

All datasets used during the current study are available from the corresponding author on reasonable request.

## Abbreviations

ADC: Apparent diffusion coefficient
DW-MRI: Diffusion-weighted magnetic resonance imaging
DWTV: Diffusion-weighted tumor volume
DWTV25 and DWTV50: Diffusion-weighted tumor volume delineated with thresholds of 25% and 50% of maximum, respectively
EANM: European Association of Nuclear Medicine
ED: Extensive disease
EGFR TKI: Epidermal growth factor receptor tyrosine kinase inhibitors
EPI: Echo-planar imaging
FDG: ^18^F-fluorodeoxy-glucose
FLT: ^18^F-fluorothymidine
GTV: Gross tumor volume
LD: Limited disease
LDH: Lactate dehydrogenase
MRI: Magnetic resonance imaging
MTV: Metabolic tumor volume
MTV41, MTV50, and MTV70: Metabolic tumor volume delineated with thresholds of 41%, 50%, and 70% of SUV_max_, respectively
NSCLC: Non-small cell lung cancer
OP-OSEM: Ordinary Poisson 3D ordered subset expectations maximization
OS: Overall survival
PET: Positron emission tomography
PFS: Progression-free survival
PS: Performance status
PTV: Proliferative tumor volume
PTV41, PTV50, PTV1.4, PTV70: Proliferative tumor volume delineated with thresholds of 40% of SUV_max_, 50% of SUV_max_, SUV = 1.4, and 70% of SUV_max_, respectively
RECIST: The Response Evaluation Criteria in Solid Tumors
RT: Radiotherapy
SCLC: Small cell lung cancer
SUV: Standardized uptake value
SUV_max_: Maximum standardized uptake value
SUV_mean_41, SUV_mean_50, and SUV_mean_1.4: Mean of SUVs included in MTV41/PTV41, MTV50/PTV50, and PTV1.4, respectively
TE: Echo time
TLG: Total lesion glycolysis
TLP: Total lesion proliferation
TR: Repetition time
