# Supplementary material for: 18F-fluorothymidine (FLT)-PET and diffusion-weighted MRI for early response evaluation in patients with small cell lung cancer: a pilot study
Source: Eur J Hybrid Imaging. 2020 Jan 27;4:2. doi: 10.1186/s41824-019-0071-5 (PMC8218141; doi:10.1186/s41824-019-0071-5)
Supplement: Supplementary file 1 — Additional file 1: Table S1. Scan data and time points [file 41824_2019_71_MOESM1_ESM.docx]

**Table S1: Scan data and time points**

| **Pt no.** | **Baseline scan** | **Project scan** | **Days from baseline FDG-PET-scan to FLT-PET/MRI** | **Days from treatment start to FLT-PET/MRI** | **FDG-PET scanner/model**  **Software**  **Reconstruction method** | **FDG-uptake interval in minutes** | **FLT-uptake interval in minutes**  **(cerebrum/thorax)** |
| --- | --- | --- | --- | --- | --- | --- | --- |
| 1 | FDG-PET/CT | FLT-PET/DW-MRI | 16 | 1 | Philips medical system/Gemini TF TOF 64  9.5.1  BLOB-OS-TF | 85 | 56/71 |
| 2 | CT | FLT-PET/DW-MRI |  | 6 |  |  | 58/76 |
| 3 | FDG-PET/CT | FLT-PET/DW-MRI | 14 | 2 | Philips medical system/Gemini TF TOF 64  9.5.1  BLOB-OS-TF | 72 | 58/76 |
| 4 | FDG-PET/CT | FLT-PET/DW-MRI | 16 | 6 | Philips medical system/Gemini TF TOF 64  9.5.1  BLOB-OS-TF | 75 | 67/84 |
| 5 | CT | FLT-PET/DW-MRI |  | 1 |  |  | 66/83 |
| 6 | FDG-PET/CT | FLT-PET/DW-MRI | 17 | 6 | Philips medical system/Gemini TF TOF 64  9.5.1  BLOB-OS-TF | 84 | 51/69 |
| 7 | FDG-PET/CT | FLT-PET/MRI  (only T1 MRI  No contrast) | 7 | 1 | Siemens/somatom definition AS_mCT  syngo.MI PET/CT 2012a  PSF+TOF 2i21s | 60 | 58/79 |
| 8 | FDG-PET/CT | FLT-PET/DW-MRI | 14 | 2 | Siemens/1094  PET/CT 2009a  PSF 3i21s | 64 | 62/84 |
| 9 | FDG-PET/CT | FLT-PET/DW-MRI | 20 | 9 | Philips medical system/Gemini TF TOF 64  9.5.1  BLOB-OS-TF | 74 | 58/77 |
| 10 | CT | FLT-PET/DW-MRI |  | 3 |  |  | 56/73 |
| 11 | FDG-PET/CT | FLT-PET/DW-MRI | 8 | 6 | Siemens/1094  PET/CT 2009a  PSF 3i21s | 62 | 54/82 |
| 12 | FDG-PET/CT | FLT-PET/DW-MRI | 21 | 6 | GE medical systems/Discovery 710  53.00  QCFX | 63 | 55/72 |
