# Supplementary material for: 18F-fluorothymidine (FLT)-PET and diffusion-weighted MRI for early response evaluation in patients with small cell lung cancer: a pilot study
Source: Eur J Hybrid Imaging. 2020 Jan 27;4:2. doi: 10.1186/s41824-019-0071-5 (PMC8218141; doi:10.1186/s41824-019-0071-5)
Supplement: Supplementary file 2 — Additional file 2: Table S2. PET- and MRI-parameters from malignant lesions [file 41824_2019_71_MOESM2_ESM.docx]

**Table S2: PET- and MRI-parameters from malignant lesions.**

| **Pt no.** | **Lesion no.** | **FDG-PET** | | **FLT-PET** | | **MRI** | **DW-MRI** | | **Voxel-by-voxel correlation coefficient** | | **Comments** |
| --- | --- | --- | --- | --- | --- | --- | --- | --- | --- | --- | --- |
|  |  | **MTV41** | **SUV_peak_** | **PTV 50** | **SUV_peak_** | **GTV** | **DWTV 25** | **ADC_median_** | **r (FDG vs. FLT)** | **r (FLT vs. DWI)** |  |
| 1 | 1-T | 23.5 | 16.5 | * | 1.6 | 45.1 | 21.3 | 1.22 | 0.50 |  |  |
|  | 1-N1 | 9.8 | 12.2 | * | 1.8 | 17.4 | 13.5 | 1.21 | 0.03 |  |  |
|  | 1-N2 | 24.3 | 13.4 | * | 1.5 | 33.9 | 30.7 | 0.90 | 0.42 |  |  |
|  | 1-N3 | 23.7 | 15.5 | * | 1.9 | 29.2 | 27.6 | 0.88 | 0.03 | 0.41 |  |
|  | 1-N4 | 17.4 | 17.3 | * | 2.1 | 13.6 | 13.5 | 1.07 | -0.24 | -0.01 |  |
|  | 1-M | 2.4 | 4.2 | 2.4 | 1.1 |  | 4.5 | 1.05 |  |  |  |
| 2 | 2-N | NA | NA | * | 1.3 | 120 | 105 | 1.54 |  | 0.08 |  |
| 3 | 3-T | 104 | 9.0 | 37.5 | 2.1 | 169 | 137 | 1.59 |  | 0.21 |  |
|  | 3-N1 | 7.2 | 8.1 | * | 1.6 | 14.2 | 9.0 | 1.96 |  | 0.07 |  |
|  | 3-N2 | 20.0 | 5.5 | * | 1.2 | 25.6 | 13.7 | 1.89 |  | -0.18 |  |
|  | 3-N3 | 27.8 | 8.0 | * | 1.3 | 49.9 | 24.3 | 1.39 |  | -0.20 |  |
| 4 | 4-T | 224 | 22.7 | 17.6 | 11.5 | 273 | 193 | 1.43 | 0.10 | -0.15 |  |
| 5 | 5-T | NA | NA | 18.7 | 2.6 | 10.4 | 17.0 | 1.74 |  | -0.02 | Previously irradiated. |
| 6 | 6-T | 4.0 | 3.9 | * | 0.6 | 1.0 | # | # |  |  |  |
|  | 6-N | 8.3 | 6.2 | 3.1 | 1.3 | 3.9 | 10.3 | 2.09 |  |  |  |
| 7 | 7-T | 94.2 | 8.3 | 15.3 | 4.0 | 149 | NA | NA | 0.49 |  |  |
|  | 7-M | 3.7 | 5.2 | 4.9 | 1.9 |  | NA | NA |  |  |  |
| 8 | 8-T1 | 51.4 | 9.7 | * | 1.7 | 57.7 | 58.9 | 1.11 |  | 0.32 | Surrounding carcinomatosis included in the tumor volumes. |
|  | 8-T2 | 2.3 | 2.0 | 0.5 | 0.6 | # | # | # |  |  |  |
|  | 8-T3 | 0.7 | 2.2 | 3.4 | 0.7 | # | # | # |  |  |  |
| 9 | 9-T1 | 125 | 12.1 | 91.2 | 1.7 | 165 | 57.1 | 1.74 |  | -0.03 | Inseparable athelectasis included in GTV. |
|  | 9-T2 |  | 3.7 | 5.0 | 1.3 | 5.3 | 3.2 | 1.01 |  | 0.42 |  |
| 10 | 10-T1 | NA | NA | 11.7 | 2.8 | 23.2 | 40.6 | 0.82 |  | -0.54 | Previously irradiated. |
|  | 10-T2 | NA | NA | 3.8 | 1.9 | 3.5 | 5.6 | 1.10 |  | -0.66 |  |
| 11 | 11-T1 | 190 | 11.8 | 74.3 | 3.0 | 285 | 167 | 1.15 | 0.50 | -0.01 |  |
|  | 11-T2 | 1.2 | 4.4 | 1.9 | 1.0 | 2.6 | 2.6 | 0.76 |  |  |  |
|  | 11-M1 | 18.6 | 8.9 | 25.0 | 2.3 | 22.1 | 24.2 | 1.03 | 0.45 | 0.12 |  |
|  | 11-M2 | 280 | 10.9 | 135 | 4.6 | 307 | 200 | 1.03 | 0.62 | -0.34 |  |
| 12 | 12-T | 15.7 | 12.8 | 15.5 | 1.2 | 20.2 | 10.5 | 1.19 |  |  |  |
|  | 12-N1 | 12.3 | 11.6 | 11.1 | 2.4 | 13.2 | 14.5 | 1.56 | 0.55 | -0.44 |  |
|  | 12-N2 | 24.8 | 10.2 | 20.9 | 2.2 | 20.4 | 18.4 | 1.85 | 0.41 | -0.16 |  |
|  | 12-N3 | 21.4 | 13.7 | 19.5 | 1.9 | 22.6 | 18.2 | 1.61 | 0.60 | -0.41 |  |

MTV41: metabolic tumor volume delineated with a threshold of 41% of SUV_max_; SUV: standardized uptake value; PTV50: proliferative tumor volume delineated with a threshold of 50% of SUV_max_; GTV: gross tumor volume; DWTV25: diffusion weighted tumor volume delineated on DW-MRI (b=800 s/mm^2^) using a threshold of 25% of maximum; ADC: apparent diffusion coefficient.

* = PTV50 could not be distinguished from background uptake; # = Tumor not visible on MRI and/or DW-MRI.

Tumor volumes in cm^2^; ADC in 10^-3^ mm^2^/s
