# Supplementary material for: 18F-fluorothymidine (FLT)-PET and diffusion-weighted MRI for early response evaluation in patients with small cell lung cancer: a pilot study
Source: Eur J Hybrid Imaging. 2020 Jan 27;4:2. doi: 10.1186/s41824-019-0071-5 (PMC8218141; doi:10.1186/s41824-019-0071-5)
Supplement: Supplementary file 3 — Additional file 3: Table S3. Comparison of PET- and MRI-parameters in lesions with response vs. no change or progression. [file 41824_2019_71_MOESM3_ESM.docx]

**Table S3: Comparison of PET- and MRI-parameters in lesions with response vs. no change or progression.** Note that no T-sites had progression, and no N-sites had no change, therefore analyses of T-sites are performed as lesions with response vs. lesions with no change, and analyses of N-sites are performed as lesions with response vs. lesions with progression. Mean difference and p-value from independent t-test. Significant results are marked with *.

|  | **Imaging modality** | **Parameter** | **n (lesion with response + lesions with no change / progression)** | **Mean (range)**  **of lesions with response** | **Mean (range)**  **of lesions with no change/progression** | **p** |  |
| --- | --- | --- | --- | --- | --- | --- | --- |
| T-sites | FDG-PET | FDG-SUV_peak_ | 9+2 | 8.0 (2.0-16.5) | 17.3 (11.8-22.7) | 0.063 |  |
|  |  | MTV41 | 8+2 | 41 (0.7-126) | 208 (191-225) | 0.002* |  |
|  |  | TLG41 | 8+2 | 312 (2-1067) | 2410 (1583-3237) | 0.006* | # |
|  | FLT-PET | FLT-SUV_peak_ | 11+3 | 1.5 (0.6-2.8) | 5.7 (2.6-11.5) | 0.007* | # |
|  |  | PTV50 | 8+3 | 21.1 (0.5-91.2) | 36.9 (17.6-74.3) | 0.472 |  |
|  |  | TLP50 | 9+3 | 35.5 (0-119) | 120 (37-171) | 0.029* |  |
|  | DW-MRI | DWTV25 | 8+3 | 41.7 (3.2-137) | 126 (17.0-193) | 0.067 |  |
|  |  | ADC_median_ | 8+3 | 1.22 (0.82-1.74) | 1.44 (1.15-1.74) | 0.315 |  |
| N-sites | FDG-PET | FDG-SUV_peak_ | 8+3 | 10.8 (5.5-17.3) | 11.8 (10.2-13.7) | 0.585 |  |
|  |  | MTV41 | 8+3 | 17.3 (7.2-27.8) | 19.5 (12.3-24.8) | 0.684 |  |
|  |  | TLG41 | 8+3 | 140 (38-275) | 168 (107-203) | 0.644 |  |
|  | FLT-PET | FLT-SUV_peak_ | 9+3 | 1.6 (1.2-2.1) | 2.2 (1.9-2.4) | 0.013* |  |
|  |  | PTV50 | 1+3 | 3.1 (3.1-3.1) | 17.2 (11.1-20.9) | 0.148 |  |
|  |  | TLP50 | 1+3 | 3.4 (3.4-3.4) | 28.6 (21-36) | 0.095 |  |
|  | DW-MRI | DWTV25 | 9+3 | 27.5 (9.0-105) | 17.0 (14.5-18.4) | 0.573 |  |
|  |  | ADC_median_ | 9+3 | 1.44 (0.88-2.09) | 1.67 (1.56-1.85) | 0.416 |  |

# Values are estimated from log-transformed data.
