# Supplementary material for: 18F-fluorothymidine (FLT)-PET and diffusion-weighted MRI for early response evaluation in patients with small cell lung cancer: a pilot study
Source: Eur J Hybrid Imaging. 2020 Jan 27;4:2. doi: 10.1186/s41824-019-0071-5 (PMC8218141; doi:10.1186/s41824-019-0071-5)
Supplement: Supplementary file 4 — Additional file 4: Table S4. FLT-uptake in normal tissue. [file 41824_2019_71_MOESM4_ESM.docx]

**Table S4: FLT-uptake in normal tissue.** Values deviating from the reference interval are written in red. References from Cysouw et al.

| **pt no.** | **FLT-PET parameters** | | | | | | | | |
| --- | --- | --- | --- | --- | --- | --- | --- | --- | --- |
|  | **Liver** | | | **Blood pool** | | | **Bone marrow** | | |
|  | **SUV_max_** | **SUV_peak_** | **SUV_mean_** | **SUV_max_** | **SUV_peak_** | **SUV_mean_** | **SUV_max_** | **SUV_peak_** | **SUV_mean_** |
| 1 | 9.4 | 8.1 | 7.4 | 1.4 | 1.2 | 1.1 | 2.1 | 1.7 | 1.6 |
| 2 | 7.8* | 5.8* | 4.8* | 0.9 | 0.8 | 0.7 | 1.4 | 1.2 | 1.1 |
| 3 | 8.1 | 7.1 | 6.1 | 1.0 | 0.7 | 0.6 | 4.2 | 3.6 | 3.6 |
| 4 | 2.5 | 2.1 | 1.8 | 0.8 | 0.7 | 0.6 | 3.4 | 2.8 | 2.5 |
| 5 | 13.0 | 11.3 | 9.7 | 1.6 | 1.3 | 1.1 | 2.7 | 2.1 | 2.1 |
| 6 | 2.3 | 2.0 | 1.6 | 0.8 | 0.7 | 0.6 | 4.8 | 4.0 | 3.9 |
| 7 | ^¤^ | ^¤^ | ^¤^ | 0.8 | 0.7 | 0.6 | 13.4 | 11.3 | 11.3 |
| 8 | 6.5 | 5.3 | 4.6 | 0.7 | 0.6 | 0.5 | 7.7 | 6.5 | 6.5 |
| 9 | 7.5 | 6.7 | 4.8 | 0.8 | 0.7 | 0.6 | 3.6 | 2.5 | 2.6 |
| 10 | 6.2 | 5.1 | 4.6 | 1.0 | 0.8 | 0.7 | 1.7 | 1.5 | 1.4 |
| 11 | 2.8 | 2.8 | 1.3 | 0.7 | 0.7 | 0.5 | 3.9 | 3.0 | 3.0 |
| 12 | ^#^ | ^#^ | ^#^ | 0.7 | 0.7 | 0.6 | 3.8 | 3.2 | 3.0 |
| Mean±SD | 6.6±3.9 | 5.5±2.9 | 4.7±2.7 | 0.93±0.29 | 0.80±0.22 | 0.68±0.20 | 4.4±3.3 | 3.6±2.8 | 3.6±2.8 |
| Reference | 3.68-8.17 | 3.46-7.46 | 3.09-6.45 | 0.47-1.12 | 0.44-1.04 | 0.38-0.93 | 5.84-12.54^ | 4.86-11.36^ | 4.41-9.74^ |

*fatty liver; ^¤^liver not included in frame; ^#^large metastases in liver; ^reference for bone marrow was based on several vertebras.
